# The Seasonal Periodicity of Healthy Contemplations About Exercise and Weight Loss: Ecological Correlational Study

**DOI:** 10.2196/publichealth.7794

**Published:** 2017-12-13

**Authors:** Kenneth Michael Madden

**Affiliations:** ^1^ Gerontology and Diabetes Research Laboratory Department of Medicine University of British Columbia Vancouver, BC Canada; ^2^ Centre for Hip Health and Mobility University of British Columbia Vancouver, BC Canada

**Keywords:** healthy lifestyle, weight loss, exercise, Internet, motivation

## Abstract

**Background:**

Lack of physical activity and weight gain are two of the biggest drivers of health care costs in the United States. Healthy contemplations are required before any changes in behavior, and a recent study has shown that they have underlying periodicities.

**Objective:**

The aim of this study was to examine seasonal variations in state-by-state interest in both weight loss and increasing physical activity, and how these variations were associated with geographic latitude using Google Trends search data for the United States.

**Methods:**

Internet search query data were obtained from Google Trends (2004-2016). Time series analysis (every 2 weeks) was performed to determine search volume (normalized to overall search intensity). Seasonality was determined both by the difference in search volumes between winter (December, January, and February) and summer (June, July, and August) months and by the amplitude of cosinor analysis.

**Results:**

Exercise-related searches were highest during the winter months, whereas weight loss contemplations showed a biphasic pattern (peaking in the summer and winter months). The magnitude of the seasonal difference increased with increasing latitude for both exercise (*R*^2^=.45, *F*_1,49_=40.09, beta=−.671, standard deviation [SD]=0.106, *P*<.001) and weight loss (*R*^2^=.24, *F*_1,49_=15.79, beta=−.494, SD=0.124, *P*<.001) searches.

**Conclusions:**

Healthy contemplations follow specific seasonal patterns, with the highest contemplations surrounding exercise during the winter months, and weight loss contemplations peaking during both winter and summer seasons. Knowledge of seasonal variations in passive contemplations may potentially allow for more efficient use of public health campaign resources.

## Introduction

One of the biggest drivers of health care costs in the United States is poor health behavior [1], and the two most associated factors with cardiovascular disease are obesity [2] and a sedentary lifestyle [3]. Attempts to promote diet and exercise are expensive; in fact, the US Government spends approximately 75 billion dollars per year in public health campaigns [4] with mixed results [5].

Traditionally, surveys have been used to examine health behaviors, but they have well-known limitations such as the tendency of respondents to answer in a socially desirable manner [6] and a long time lag between data collection and analysis [7]. A new technique, referred to as “Infodemiology,” allows researchers to look into the motivations of patients through the use of open access records of Internet search activity [8,9]. As Google has made their search data accessible to the public, analysis of these data allows a glimpse into a population’s hidden healthy contemplations, an area that is difficult to measure with standard surveys [7]. This open access search data allow researchers to undertake a new type of ecological correlational study [6]. Ecological studies that examine the association between variables at a population level have a long history in the generation of hypotheses for future research [10]. This study looks for population-wide associations between Google search data and latitude to look for potential seasonal and geographic patterns in healthy contemplations.

The real-time nature of Internet search data [7] allows new investigations into the periodicity of healthy contemplations. A recent study has examined the day of the week that is the “healthiest day” [11] and the day on which more people are contemplating smoking cessation [12]. The seasons also have an effect on healthy contemplations; interest (as measured by Internet search activity) on mental health conditions peaks during the winter months [13], whereas searches for information on restless leg syndrome [14] and urinary tract infections [15] peak during the summer months. Healthy behaviors such as healthy eating [16] and increased physical activity [17] show strong variations with the seasons. Low levels of vitamin D also show strong seasonal variability [18], and this variability increases with increasing northerly latitude [19]. Given the well-established associations between both obesity [19] and low levels of outdoor activity [20] with vitamin D levels, it suggests that contemplations around weight loss and exercise may also show both seasonal (winter vs summer) and geographic (increasing northern latitude) patterns. Discovering the periodicity of healthy contemplations allows us insight into the otherwise hidden thoughts of populations and is one potential method of more accurately timing public health education initiatives.

This study seeks to examine the seasonality of passive healthy contemplations on a state-by-state basis with respect to weight loss and exercise by examining the relationship between the seasonal variations in Google searches for these terms and geographic latitude. As a previous study has suggested that physical activity [17] and healthy eating [16] increase in the summer months, we hypothesized that healthy contemplations surrounding increasing physical activity would show much more seasonality than those surrounding weight loss.

## Methods

### Internet Search Data

Google Trends is a Web-based tool that can compute how many searches have been performed for any given keyword or combination of keywords. This system automatically normalizes search activity for overall search activity to a score between 0 and 100 [21]. Search activity can be narrowed to any given country and state within a country. In keeping with the current standards for reporting Google Trends data [22], search data were obtained in weekly intervals from 2004 to 2016, the database was accessed on August 17, 2016, and the complete text is reported below. As this study only uses publicly available aggregate data, approval from human subjects ethics board was deemed unnecessary.

As in previously published studies, all search terms using search data in the public health field [11-13] were chosen systematically before starting any data analysis and are shown below. We initially started with two initial search terms: “exercise” and “weight loss.” Each of these terms was entered into the keyword search tool [21], a Web-based application (Alphabet Inc.) that suggests keywords commonly related to any entered keyword and the normalized search activity associated with it. All related keywords with a higher search volume were added to our list, and each new keyword was in turn entered into the keyword search tool until no new search terms were located. Our eventual keyword lists for interest in weight loss and exercise were entered together into Google Trends using logical “or” operators. Our total keywords consisted of the following:

- *Exercise keywords*: “exercise,” “how to exercise,” “exercise more,” “exercises,” or “do more exercise.”
- *Weight loss keywords*: “weight loss,” “how to lose weight,” “lose weight,” “losing weight,” “weight loss diet,” “weight loss plan,” “diet plan,” or “diet meal plan.”

### Analysis of Seasonality

The magnitude of the seasonal shifts in Internet searches for both weight loss (DeltaWeight) and exercise (DeltaExercise) was initially determined by the difference between the average volume of searches in winter months (December, January, and February) and summer months (June, July, and August) as done in previous studies [13,15].

Seasonal variation in search activity was also obtained using cosinor analysis [23]. Cosinor analysis uses the entire year’s dataset, has been used previously with Google Trends data [14], and uses a parametric seasonal model in which a sinusoid is fit to an observed time series as part of a generalized linear model [23]. Specifically, cosinor analysis provides an assessment of the amplitude, which is a measure of the magnitude of the seasonal variation in the data [23]. Amplitude was determined for search terms related to weight loss (weight loss amplitude) and exercise (exercise amplitude). Cosinor analyses were performed using the season package in R version 3.1.0 (R Project) [24] and were determined using weekly running normalized search data for our total keywords from 2004 to 2016. Latitudes for the center of each state were obtained from the US Department of Commerce [25].

As opposed to seasonality, the average weekly search activity was determined by averaging the number of exercise (MeanExercise)- and weight loss-related (MeanWeight) searches over the entire 2004 to 2016 period.

### Statistical Analysis

Our primary response variables were DeltaWeight, DeltaExercise, exercise amplitude, weight loss amplitude, MeanExercise, and MeanWeight on a state-by-state basis. Our predictor variable was the latitude of the center of each state. Scatterplots were visually inspected for outlier data, and density plots were examined to identify data skewing. Any predictor that demonstrated skewing was logarithmically transformed (base ten) before both univariate and multivariate analyses [26]. Plots of residuals and a quantile-quantile plot were examined for each model. For each simple linear regression, the *F* statistic, the coefficient of determination (*R*^2^), degrees of freedom, and standardized beta coefficients are reported [26]. The R core software package version 3.0.1 (The R Project for Statistical Computing) was used for statistical analysis with a significance level of *P*<.05 [25].

## Results

### Overall Seasonal Search Patterns

**Figure 1:**
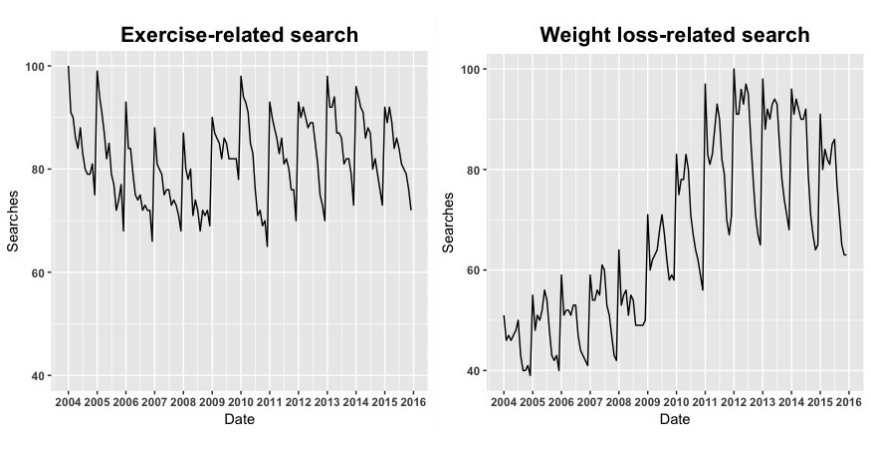
The seasonal changes in healthy contemplations with respect to exercise and weight loss using Google Trends. Google Trends normalizes search activity for overall search activity to a score between 0 and 100.

Weekly exercise-related searches in the United States had a mean of 79.6 (SD 0.3), and weight loss-related searches had a mean of 59.2 (SD 0.7). As shown in Figure 1, interest in exercise was highest during winter months, dropping off steadily afterwards. For the entire United States, exercise-related search activity was 91.4 (SD 1.3) during winter months, falling to 80.8 (SD 1.5) during summer months. Weight loss searches demonstrated a biphasic response, with peak search observed during winter (72.7 [SD 5.3]) and summer (70.3 [SD 5.2]) months.

### Latitude and Seasonality of Exercise Searches

**Figure 2:**
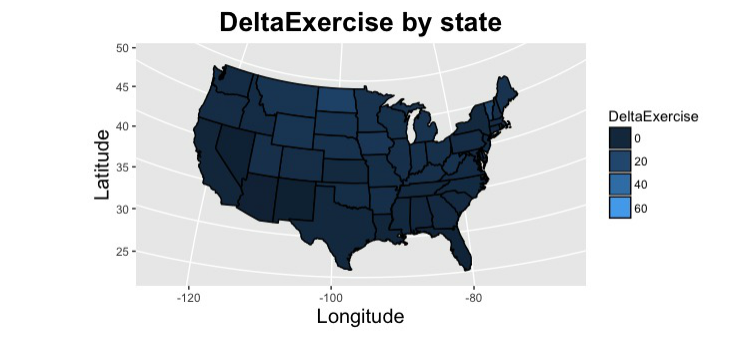
The seasonal difference in healthy contemplations surrounding exercise are shown by state. Seasonal difference was defined as the normalized number of searches in winter months (December, January, and February) minus the number in summer months (June, July, and August). The lighter blue color corresponds with an increase in seasonal variation.

**Figure 3:**
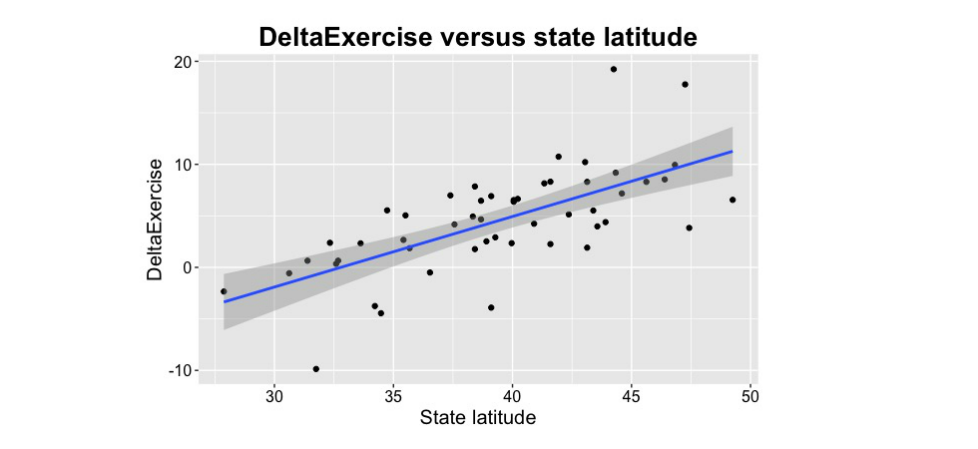
The increasing seasonal difference in healthy contemplations surrounding exercise with increasingly northern latitudes is demonstrated. Seasonal difference was defined as the normalized number of searches in winter months (December, January, and February) minus the number in summer months (June, July, and August).

Seasonal peaks and troughs were more pronounced in states geographically situated at southern latitudes versus those located further to the south (Figure 2). The average number of normalized weekly searches related to exercise (*R*^2^=.07, *F*_1,49_=3.45, beta=−.256, SD=0.014, *P*=.07) did not demonstrate a statistically significant correlation with state latitude. However, the seasonal difference (DeltaExercise) in exercise-related searches increased at higher latitudes (Figure 3). The correlation between seasonal differences and latitude was demonstrated by both DeltaExercise (*R*^2^=.45, *F*_1,49_=40.09, beta=−.671, SD=0.106, *P*<.001) and with the cosinor analysis (*R*^2^=.16, *F*_1,49_=9.37, beta=.401, SD=0.131, *P*=.004).

### Latitude and Seasonality of Weight Loss Searches

Similar to the exercise search results, the average number of searches related to weight loss (*R*^2^=.07, *F*_1,49_=3.78, beta=−.268, SD=0.138, *P*=.06) did not show a significant correlation with latitude. With respect to weight loss-related search activity, DeltaWeight showed a significant correlation with state latitude (*R*^2^=.24, *F*_1,49_=15.79, beta=−.494, SD=0.124, *P*<.001). However, the cosinor analysis did not show any statistically significant relationship between state latitude and amplitude (*R*^2^=.015, *F*_1,49_=0.73, beta=−.122, SD=0.142, *P*=.40).

## Discussion

### Principal Findings

Healthy contemplations surrounding exercise (as measured by Internet search activity) showed a peak during the winter months as compared with the summer months. Exercise-related searches also showed a strong state-by-state seasonality, showing more seasonal differences with increasing northern latitude. Unlike healthy contemplations around exercise, weight loss search activity demonstrated a biphasic pattern, peaking in both winter and summer months.

### Previous Work

It has been well established that physical activity shows strong seasonal variations. Both African American and white children show higher levels of vitamin D in the summer, likely because of increased outdoor physical activity [27]. Objective accelerometer measures have demonstrated higher levels of physical activity in adult women [17], patients with chronic obstructive lung disease [28], and older adults [29,30] during the summer months. Physical activity as measured by a 7-day patient recall of adults aged 20 to 70 years has also shown similar peaks in physical activity during the summer months [31]. Although health behaviors were not measured in this study, our findings demonstrate that contemplations about physical activity do not necessarily parallel health behavior patterns; Internet searches surrounding exercise were shown to peak in the winter as opposed to summer months, and this seasonality increased with increasing latitude.

Previous examination of the seasonality of healthy eating behaviors has shown conflicting results. Dietary log data have suggested that dietary intake patterns have remained stable across all seasons [32,33]. However, a longitudinal examination of the Healthy Eating Index in women aged 40 to 60 years demonstrated less healthy diets during the winter [34]. Markers of healthy eating, such as salad [35] and vegetables [16], are more frequently eaten during the spring and summer months. A more detailed examination of dietary components has shown that both fat and caloric intake are highest during autumn months, but carbohydrate intake is highest in the spring [31]. The seasonality of caloric intake has also been supported by a worldwide correlation between satellite measures of artificial light and obesity [36]. Although this study did not examine dietary behaviors, we were able to demonstrate a biphasic (summer and winter) peak in weight loss contemplations, which diverge from previously established patterns of health behaviors.

Other investigations have examined the periodicity of healthy considerations over various different time scales. A previous analysis of Google Trends data has shown that many healthy contemplations seem to peak earlier in the week (Mondays) as shown for both smoking cessation [12] and searches for the word “healthy” [11]. This weekly pattern suggests that individuals may be more susceptible to public health messaging earlier in the week [11,12]. Our results suggest that the periodicity of healthy contemplations follow a seasonal cycle, as well as a circaseptan cycle, with increased contemplations about exercise occurring in winter months and increased contemplations surrounding weight loss occurring in both winter and summer months.

### Clinical Implications and Potential Mechanisms

Our Google Trends measures of healthy contemplations have demonstrated that searches for both exercise and weight loss follow specific seasonal patterns. This suggests that at certain times of the year, populations might be more susceptible to public awareness campaigns, both for exercise (winter months) and weight loss (winter and summer months), and that this greater susceptibility is even larger in more northerly states. Knowledge of seasonal variations in passive contemplations may allow for more effective use of public health campaign resources, although this requires further study. Our study also demonstrated that seasonal patterns in *healthy contemplations* do not necessarily parallel previously demonstrated seasonal patterns in *healthy behaviors*.

The peak in interest in both exercise and weight loss in the winter months may be explained by the tradition of forming New Year’s resolutions; 50% of all Americans participate in this tradition and the most common resolutions involve healthy behaviors [37]. In addition, the fact that interest in both exercise and weight loss showed a larger seasonal shift with increasing latitude indicates that there may be underlying physiological mechanisms behind this pattern. The winter months are characterized by more exposure to artificial light sources [38], resulting in decrease in melatonin production in humans [39]. There is a well-documented relationship between decreased melatonin levels and increasing weight [40]; in fact, melatonin administration in both human [41] and animal studies [42] results in weight loss. This increased weight gain in the winter months may explain both the observed increase in healthy contemplations and the exacerbation of this seasonality in northern states. The peak in weight loss searches during the summer months may be because of the differences in summer as opposed to winter clothing; previous motivational surveys have demonstrated that appearance is the second most common motivating factor for losing weight [43].

### Limitations and Future Research

Although this study is suggestive with respect to the seasonal timing of healthy contemplations, Internet searches do not necessarily indicate an intent to pursue healthy behaviors, given that the context underlying each search is not known. Even without this underlying context, a previous study examining tobacco products has suggested that online search behavior can predict offline behavior [44]. It remains to be investigated whether timing public health awareness campaigns to coincide with the seasonal patterns noted in this study will increase their efficacy. In addition, Google Trends only provides normalized results of search data as opposed to absolute number of searches. Offsetting this, however, is the fact that the number of keyword searches for diet and exercise number in the billions [45]; thus, any seasonal increase in normalized results likely represents millions of additional searches.

### Conclusions

Healthy contemplations follow specific seasonal patterns, with highest contemplations surrounding exercise during the winter months, and weight loss contemplations peaking during both winter and summer seasons.

## Abbreviations

SD: standard deviation
